# Supplementary material for: Analysis of Stepped‐Wedge Cluster Randomized Trials When Treatment Effects Vary by Exposure Time or Calendar Time
Source: Stat Med. 2025 Sep 24;44(20-22):e70256. doi: 10.1002/sim.70256 (PMC12459233; doi:10.1002/sim.70256)
Supplement: Supplementary file 1 — Data S1. Supporting Information. [file SIM-44-0-s001.pdf]

A Appendix: Data-generating process for SW-CRTs with time-varying treatment effects

We use a model-based approach to describe the underlying data-generating process of cross-sectional SW-CRTs and to define the time-averaged treatment effect estimands of interest. In a SW-CRT with fixed cluster-period cell sizes, we can specify potential outcomes $Y_{ijk}(q)$ for individual $k \in \{1, ..., K\}$ in time period $j \in \{1, ..., J\}$ of cluster $i \in \{1, ..., I\}$, randomized to sequence $q \in \{1, ..., Q\}$, where the treatment in sequence q is introduced during period $j = q + 1$ in a SW-CRT. Note that there are typically $Q = J - 1$ sequences in a standard complete SW-CRT design, with I/Q clusters equally randomized into each sequence q.

We can then describe a structural model for the generalized conditional expected value of the potential outcome $Y_{ijk}(q)$ for individual k observed in period j of cluster i in sequence q. The model is based on the generic structural model outlined in Li et al. [9] and adapted in Kenny et al. [3]:

$$g(E[Y_{ijk}(q)|\Gamma_j, C_{qijk}]) = \Delta_{qij} + \Gamma_j + C_{qijk} \tag{12}$$

where $g(.)$ is a link function, Δ_{qij} is the treatment effect structure, Γ_j is the calendar time trend, and C_{qijk} is the cluster-specific, time-specific, and/or individual-specific heterogeneity term which captures the correlation structure of the data [9]. Treatment effect structures Δ_{qij} are unique to each randomization sequence q, therefore we do not have to condition on the treatment effect structure Δ_{qij} in equation 12.

Throughout the current article, we focus on models with an identity link function. Let Y_{ijk} denote the observed outcome for individual k in period j of cluster i. If we assume that clusters are assigned to sequences randomly (*exchangeability*) and if cluster i is randomized to sequence q (*consistency*), then $E[Y_{ijk}(q)|\Gamma_j, C_{ijk}]$ being the conditional expectation of the potential outcome $Y_{ijk}(q)$ is identified by $E[Y_{ijk}|\Delta_{ij}, \Gamma_j, C_{ijk}]$ being the expectation of the observed outcome Y_{ijk} conditioned on the treatment effect structure Δ_{ij}. By conditioning on the treatment effect structure, calendar time trend, and specific departures from the marginal average, we have:

$$E[Y_{ijk}|\Delta_{ij}, \Gamma_j, C_{ijk}] = \Delta_{ij} + \Gamma_j + C_{ijk} \tag{13}$$

with the assumptions of exchangeability and consistency typically holding in a randomized trial. In equation 13, the subscripts for sequence q are removed since they are determined by i and j given Δ_{ij}.

As mentioned in the introduction, most statistical models used to analyze data from stepped wedge designs assume an immediate treatment effect. In the current work, we additionally assume that the treatment effect may vary as a function of exposure time s or calendar time period j (but not both). We accordingly rewrite

equation 13 as:

$$E[Y_{ijk}|\dot{X}_{ij},\Gamma_j,C_{ijk}] = \dot{X}_{ij}\theta + \Gamma_j + C_{ijk}\;, \tag{14}$$

$$E[Y_{ijk}|\ddot{X}'_{ij},\Gamma_j,C_{ijk}] = \ddot{X}'_{ij}\delta + \Gamma_j + C_{ijk}\;, \tag{15}$$

$$E[Y_{ijk}|\dddot{X}'_{ij},\Gamma_j,C_{ijk}] = \dddot{X}'_{ij}\xi + \Gamma_j + C_{ijk}\;. \tag{16}$$

Where the expected outcome Y_{ijk} for individual k observed in period j of cluster i is now conditional on $\dot{X}_{ij}$, $\ddot{X}'_{ij}$, or $\dddot{X}'_{ij}$ being the immediate, exposure time-varying, or calendar time-varying treatment effect structures, respectively.

Equation 14 represents a general model with an immediate treatment effect structure. Where q_i is the sequence q containing a given cluster i, then $\dot{X}_{ij} = I(j > q_i)$ is the indicator (= 1 when receiving treatment, or = 0 when receiving control) for the (single) immediate treatment effect θ for the individuals observed during period j of cluster i (equation 14).

Equation 15 represents a general model with an exposure time-varying treatment effect structure. We define $s_{ij} = \max(j - q_i, 0)$ as the amount of exposure time periods $s \in \{0, ..., J-1\}$ that individuals k in period j of cluster i have been receiving the treatment (where $s = 0$ if receiving the control). The indicator for exposure time s being a given $t > 0$ periods is then $I(s_{ij} = t) = I(j - q_i = t)$. Accordingly, $\ddot{X}'_{ij} = (I(s_{ij} = 1), I(s_{ij} = 2), ..., I(s_{ij} = J-1))$ is the 1 by $J-1$ row vector of indicators corresponding to $\delta = (\delta_1, \delta_2, ..., \delta_{J-1})'$ as the $J-1$ by 1 column vector of the different exposure time-varying treatment effects δ_s for individuals observed during period j of cluster i.

Finally, equation 16 represents a general model with a calendar time-varying treatment effect structure. Accordingly, $\dddot{X}'_{ij} = (I(j = 2 \;\&\; j > q_i), I(j = 3 \;\&\; j > q_i), ..., I(j = J \;\&\; j > q_i))$ is the 1 by $J-1$ row vector of indicators corresponding to $\xi = (\xi_2, \xi_3, ..., \xi_J)'$ as the $J-1$ by 1 column vector of the different calendar time-varying treatment effects ξ_j for individuals observed during period j of cluster i.

Notably, equation 14 is a special case of equations 15 and 16, where $\delta_s = \theta \,\forall\, s$ and $\xi_j = \theta \,\forall\, j$, respectively.

To help further clarify, we include examples below of the vectors and matrices denoting the different time-varying treatment effect structures and other model components in a 3 cluster, 4 period SW-CRT. Assuming a fixed K individuals in each cluster-period cell, we can specify:

$$\bar{Y}_{ij} = \frac{\sum_{k=1}^{K} Y_{ijk}}{K}$$

$$Y_i = (\bar{Y}_{i1}, ..., \bar{Y}_{iJ})'$$

$$Y = (Y_1', ..., Y_I')'$$

with Y being the complete vector of cluster-period cell mean outcomes for all cluster-period cells. Then, we

correspondingly specify:

$$X_i = (X_{i1}, ..., X_{iJ})'$$

$$X = (X_1', ..., X_I')'$$

with X being the general complete treatment effect structure ($\dot{X}, \ddot{X}, \dddot{X}$ being specific examples of which) for all cluster-period cells in a given trial.

In the example of a 3 cluster, 4 period SW-CRT, we then have the following immediate, exposure time-varying, and calendar time-varying treatment effect structures:

$$\dot{X} = \begin{pmatrix} 0 \\ 1 \\ 1 \\ 1 \\ - \\ 0 \\ 0 \\ 1 \\ 1 \\ - \\ 0 \\ 0 \\ 0 \\ 1 \end{pmatrix}, \ddot{X} = \begin{pmatrix} 0 & 0 & 0 \\ 1 & 0 & 0 \\ 0 & 1 & 0 \\ 0 & 0 & 1 \\ - & - & - \\ 0 & 0 & 0 \\ 0 & 0 & 0 \\ 1 & 0 & 0 \\ 0 & 1 & 0 \\ - & - & - \\ 0 & 0 & 0 \\ 0 & 0 & 0 \\ 0 & 0 & 0 \\ 1 & 0 & 0 \end{pmatrix}, \dddot{X} = \begin{pmatrix} 0 & 0 & 0 \\ 1 & 0 & 0 \\ 0 & 1 & 0 \\ 0 & 0 & 1 \\ - & - & - \\ 0 & 0 & 0 \\ 0 & 0 & 0 \\ 0 & 1 & 0 \\ 0 & 0 & 1 \\ - & - & - \\ 0 & 0 & 0 \\ 0 & 0 & 0 \\ 0 & 0 & 0 \\ 0 & 0 & 1 \end{pmatrix}$$

with each row corresponding to a different cluster-period cell, and dashed lines separating the 3 clusters. The above treatment effect structure correspond to an immediate treatment effect θ, exposure time-varying treatment effects $(\delta_1, \delta_2, \delta_3)'$, and calendar time-varying treatment effects $(\xi_2, \xi_3, \xi_4)'$, respectively.

Finally, where Γ_j is the true underlying calendar time trend at period j, the calendar time trend described earlier in the data-generating model can also be specified as:

$$\Gamma = 1_I \otimes (\Gamma_1, ..., \Gamma_J)'.$$

We define 1_n as an n-length column vector of 1's and $\otimes$ denotes the Kronecker product. Then Γ is subsequently

modeled in the analytic models with fixed period indicators, denoted as:

$$P = 1_I \otimes (P_1', ..., P_J')' = 1_I \otimes \mathbb{I}_J$$

where P_j is the 1 by J row vector of indicators for each period j (i.e., given time $j = c$ where $1 < c < J$, then $P_c = (I(j = 1), ..., I(j = c - 1), I(j = c), I(j = c + 1), ..., I(j = J)) = (0, ..., 0, 1, 0, ..., 0))$. Altogether, P is the complete period structure for all cluster-period cells, with $\mathbb{I}_n$ denoting an n by n identity matrix.

Altogether, with $Z = (X, P)$ and $V = \mathbb{I}_I \otimes R_i$ (where $\mathbb{I}_I$ is an I by I dimension identity matrix), then the analytic mixed effects model point estimator is $\hat{\beta} = (Z'V^{-1}Z)^{-1}ZV^{-1}Y$.

B Appendix: Proof of Theorem 1

Derivation of the IT estimator when the treatment effect varies with calendar time.

Assuming the true underlying marginal model has calendar time-varying treatment effects:

$$E[\bar{Y}_{ij}|\dddot{X}'_{ij}, P_j] = \dddot{X}'_{ij}\xi + P_j\phi\,,$$

we can use equation 7 to demonstrate that the expected value of the immediate treatment effect is:

$$E[\widehat{IT}|\dddot{X}'_{ij}, P_j] = \frac{12(1+\gamma Q)}{Q(Q+1)(\gamma Q^2 + 2Q - \gamma Q - 2)} \sum_{j=1}^{J}\sum_{q=1}^{Q}\left[Q(I(j>q)) + 1 - j + \frac{\gamma Q(2q-Q-1)}{2(1+\gamma Q)}\right] E[\bar{Y}_{qj}|\dddot{X}'_{qj}, P_j]$$

summed across sequences $q \in \{1, ..., Q\}$ and periods $j \in \{1, ..., J\}$. Since (1.) $\xi_1 = \xi_J = 0$, and (2.) the treatment effect structure of cluster i is fully determined by the sequence q it is randomized into, allowing us to rewrite $\dddot{X}'_{ij}\xi = \dddot{X}'_{qj}\xi = I(j>q)\xi_j$ (with $I(j>q)$ referring to an indicator for whether the index for a given period j is higher than that of a given sequence q), then the above equality can be written as:

$$E[\widehat{IT}|\dddot{X}'_{ij}, P_j] = M\sum_{j=2}^{J-1}\sum_{q=1}^{Q}\left[Q(I(j>q)) + 1 - j + \frac{\gamma Q(2q-Q-1)}{2(1+\gamma Q)}\right](I(j>q)\xi_j + P_j\phi))\,,$$

where

$$M \equiv \frac{12(1+\gamma Q)}{Q(Q+1)(\gamma Q^2 + 2Q - \gamma Q - 2)}\,.$$

In Kenny et al. [3], the following equality was shown to hold:

$$\sum_{j=1}^{J}\sum_{q=1}^{Q}\left[Q(I(j>q)) + 1 - j + \frac{\gamma Q(2q-Q-1)}{2(1+\gamma Q)}\right] P_j\phi = 0\,.$$

Therefore, we can write:

$$\begin{aligned}
E[\widehat{IT}|\dddot{X}'_{ij}, P_j] &= M\sum_{j=2}^{J-1}\sum_{q=1}^{Q}\left[Q(I(j>q)) + 1 - j + \frac{\gamma Q(2q-Q-1)}{2(1+\gamma Q)}\right] I(j>q)\xi_j \\
&= M\sum_{j=2}^{J-1}\sum_{q=1}^{j-1}\left[Q + 1 - j + \frac{\gamma Q(2q-Q-1)}{2(1+\gamma Q)}\right]\xi_j \\
&= M\sum_{j=2}^{J-1}\left[(j-1)(Q+1-j) + \frac{\gamma Q}{2(1+\gamma Q)}\sum_{q=1}^{j-1}(2q-Q-1)\right]\xi_j\,.
\end{aligned}$$

Since $\sum_{q=1}^{j-1} q = \frac{j(j-1)}{2}$, it holds that:

$$E[\widehat{IT}|\dddot{X}'_{ij}, P_j] = M\sum_{j=2}^{J-1}\left[(j-1)(Q+1-j) + \frac{(\gamma Q)(j-1)}{2(1+\gamma Q)}(-Q-1+j)\right]\xi_j.$$

Altogether, the immediate treatment estimator is in expectation a weighted average of the calendar time-varying treatment effect estimands ξ_j:

$$
\begin{aligned}
E[\widehat{IT}|\dddot{X}'_{ij}, P_j] &= \frac{12(1+\gamma Q)}{Q(Q+1)(Q-1)(\gamma Q+2)} \sum_{j=2}^{J-1} \left[(j-1)(Q+1-j) \left(\frac{2+\gamma Q}{2+2\gamma Q} \right) \right] \xi_j \\
&= \sum_{j=2}^{J-1} \frac{6(j-1)(Q+1-j)}{Q(Q+1)(Q-1)} \xi_j \,.
\end{aligned}
$$

This concludes the proof. □

Notably, Theorem 1 and the rest of the subsequent derivations are done with generalized least squares, assuming the true variance components τ_α^2, τ_ω^2, σ_e^2, and $\gamma = \frac{\tau_\alpha^2}{\tau_\alpha^2+\tau_\omega^2+\sigma_e^2/K}$ are known. If implemented with feasible generalized least squares, using the estimated variance components $\hat{\tau}_\alpha^2$, $\hat{\tau}_\omega^2$, $\hat{\sigma}_e^2$, and $\hat{\gamma} = \frac{\hat{\tau}_\alpha^2}{\hat{\tau}_\alpha^2+\hat{\tau}_\omega^2+\hat{\sigma}_e^2/K}$, the results can still hold across values of the estimated $\hat{\gamma}$ instead of the true γ, as shown here. These results hold for linear mixed effects models with either an independence ($\gamma = 0$), exchangeable ($\gamma = \frac{\tau_\alpha^2}{\tau_\alpha^2+\sigma_e^2/K}$), or nested exchangeable ($\gamma = \frac{\tau_\alpha^2}{\tau_\alpha^2+\tau_\omega^2+\sigma_e^2/K}$) correlation structure.

Crucially, the weighting of the calendar time-varying treatment effect estimands in the immediate treatment estimator is not dependent on γ. Theorem 1 still holds regardless of whether generalized least squares or feasible generalized least squares was used, as the immediate treatment effect estimator is shown above to be a weighted sum of the true calendar time-varying treatment effect estimands, and do not depend on the variance components, known or unknown.

C Appendix: Behavior of the ETATE estimator with a true underlying calendar time-varying treatment effect structure

Derivation of the ETATE estimator when the treatment effect varies with calendar time

The ETI mixed effects model (equation 5):

$$Y_{ijk} = \ddot{X}'_{ij}\delta + P_j\phi + \alpha_i + \omega_{ij} + \epsilon_{ijk} \, ,$$

can be re-written with matrix notation using the following design matrix $\ddot{Z} = (\ddot{X}, P)$, and vector of coefficients $\beta_{ETI} = (\delta', \phi')'$:

$$Y = \ddot{Z}\beta_{ETI} + v \, .$$

We can specify this above formula with cluster-period means, with Y being the vector of cluster-period mean outcomes $\bar{Y}_{ij}$ over individuals in period $j \in \{1, ..., J\}$ of cluster $i \in \{1, ..., I\}$, and v being the vector of heterogeneity terms $v_{ij} = \alpha_i + \omega_{ij} + \epsilon_{ij}$ on the cluster-period cell level, where $v \overset{iid}{\sim} N(0, V)$. In such a mixed effects model with a nested exchangeable correlation structure, $V = \mathbb{I}_I \otimes R_i$ is the IJ by IJ block diagonal variance-covariance matrix of Y (where $\mathbb{I}_I$ is an I by I dimension identity matrix) and:

$$R_i = Var(\bar{Y}_{ij})(\mathbb{I}_J(1-\gamma) + \mathbb{J}_j(\gamma))$$

(where $\mathbb{I}_J$ and $\mathbb{J}_J$ are J by J dimension matrices, representing the identity matrix and a matrix of ones, respectively), and where $\otimes$ represents the Kronecker product. With a nested exchangeable correlation structure and fixed cluster-period sizes K, $\gamma = \frac{\tau^2_\alpha}{\tau^2_\alpha + \tau^2_\omega + \sigma^2_e/K}$, and $Var(\bar{Y}_{ij}) = Var(v_{ij}) = \tau^2_\alpha + \tau^2_\omega + \sigma^2_e/K$ cancels out of the generalized least squares (GLS) point estimator.

The GLS point estimator for the ETI mixed effects model can accordingly be written as:

$$\widehat{\beta}_{ETI} = (\ddot{Z}'V^{-1}\ddot{Z})^{-1}\ddot{Z}'V^{-1}Y \, .$$

C.1 Derivation of the ETATE estimator when the treatment effect varies with calendar time in a standard Q sequence, J period SW-CRT design

Here, we outline how the ETATE estimator can be written as a weighted average of the CTI estimands in a simple and standard SW-CRT design with I clusters and J periods, where there is $I/Q = 1$ cluster in each randomization sequence $q \in \{1, ..., Q\}$ and $I = J - 1$. This yields an equivalent point estimator to scenarios with Q sequences with equal allocation of $I/Q > 1$ clusters randomized into each sequence q.

We must first derive the ETI point estimators $\hat{\delta}_s$ where $s \in \{1, .., J-1\}$. To do so, we first derive

$(\ddot{Z}'V^{-1}\ddot{Z})$ with block matrices, such that:

$$(\ddot{Z}'V^{-1}\ddot{Z}) = \frac{1}{\sigma^2 + \tau_\alpha^2 + \tau_\omega^2} \begin{pmatrix} \Omega_{11} & \Omega_{12} \\ \Omega_{21} & \Omega_{22} \end{pmatrix}$$

where $\sigma^2 = \frac{\sigma_e^2}{K}$. Here, Ω_{11} is the top-left $(J-1)$ by $(J-1)$ block matrix corresponding with the ETI effects δ_s, and Ω_{22} is the bottom-right J by J matrix corresponding with the time period effects ϕ_j. Crucially, $(\ddot{Z}'V^{-1}\ddot{Z})$ using information from all observed cluster-period cells is a positive-definite matrix; hence we can define:

$$(\ddot{Z}'V^{-1}\ddot{Z})^{-1} = (\sigma^2 + \tau_\alpha^2 + \tau_\omega^2) \begin{pmatrix} \Omega_{11} & \Omega_{12} \\ \Omega_{21} & \Omega_{22} \end{pmatrix}^{-1} .$$

The first $J-1$ rows of $(\ddot{Z}'V^{-1}\ddot{Z})^{-1}$ corresponding to the ETI effects is then equal to:

$$(\ddot{Z}'V^{-1}\ddot{Z})^{-1}_{[\delta_1,\ldots,\delta_{J-1}]} = (\sigma^2 + \tau_\alpha^2 + \tau_\omega^2) \begin{pmatrix} (\Omega_{11} - \Omega_{12}\Omega_{22}^{-1}\Omega_{21})^{-1} & -(\Omega_{11} - \Omega_{12}\Omega_{22}^{-1}\Omega_{21})^{-1}\Omega_{12}\Omega_{22}^{-1} \end{pmatrix} .$$

Subsequently, $(\ddot{Z}'V^{-1}\ddot{Z})^{-1}_{[\delta_1,\ldots,\delta_{J-1}]}\ddot{Z}'V^{-1}$ is the $(J-1)$ by $J(J-1)$ matrix:

$$(\ddot{Z}'V^{-1}\ddot{Z})^{-1}_{[\delta_1,\ldots,\delta_3]}\ddot{Z}'V^{-1} = \begin{pmatrix} \lambda_{[\delta_1]} \\ \vdots \\ \lambda_{[\delta_{J-1}]} \end{pmatrix}$$

where $\lambda_{[\delta_s]}$ is the row vector corresponding to coefficient δ_s in $(\ddot{Z}'V^{-1}\ddot{Z})^{-1}_{[\delta_1,\ldots,\delta_{J-1}]}\ddot{Z}'V^{-1}$, with $J(J-1)$ entries of $\lambda_{[\delta_s]ij}$ corresponding to cluster-period mean observations from period $j \in \{1, ..., J\}$ of cluster $i \in \{1, ..., I\}$, with $I = J - 1$.

Finally, $\hat{\delta}$, the resulting vector of the ETI estimators is:

$$\hat{\delta} = (\ddot{Z}'V^{-1}\ddot{Z})^{-1}_{[\delta_1,\ldots,\delta_{J-1}]}\ddot{Z}'V^{-1}Y .$$

Recall that Y is the vector of cluster-period cell means $\bar{Y}_{ij}$. As a result, we can write each $\hat{\delta}_s$ as:

$$\hat{\delta}_s = \sum_{i=1}^{I}\sum_{j=1}^{J} \lambda_{[\delta_s]ij}\bar{Y}_{ij} .$$

Assuming the underlying marginal model has calendar time-varying treatment effects, such that:

$$E[\bar{Y}_{ij}|\dddot{X}'_{ij}, P_j] = \dddot{X}'_{ij}\xi + P_j\phi$$

is true, the conditional expectation of a given ETI estimator $\hat{\delta}_s$, given covariates $\dddot{X}'_{ij}$ for the true CTI treatment effect structure is then:

$$E[\hat{\delta}_s|\dddot{X}'_{ij}, P_j] = \sum_{i=1}^{I}\sum_{j=1}^{J} \lambda_{[\delta_s]ij} E[\bar{Y}_{ij}|\dddot{X}'_{ij}, P_j]$$
$$= \sum_{i=1}^{I}\sum_{j=1}^{J} \lambda_{[\delta_s]ij} (\dddot{X}'_{ij}\xi + P_j\phi)\,.$$

In Kenny et al. [3], the following equality was shown to hold:

$$\sum_{i=1}^{I}\sum_{j=1}^{J} \lambda_{[\delta_s]ij}(P_j\phi) = 0\,.$$

Furthermore, we assume with a true underlying calendar time-varying treatment effect, (1.) $\xi_1 = \xi_J = 0$, because the first and last periods ($j = 1$ and $j = J$) have no within-period variation in treatment effect (being unexposed and all exposed to treatment, respectively). As a result, the observations from these periods do not contribute to $E[\hat{\delta}_s|\dddot{X}'_{ij}, P_j]$. Therefore, we sum over periods $j \in [2, J-1]$, and observe that within these periods, only clusters $i \in [1, j-1]$ receive the corresponding calendar-time varying treatment ξ_j. Additionally, rewriting (2.) $\dddot{X}'_{ij}\xi = I(j > i)\xi_j$ (with $I(j > i)$ referring to an indicator for whether the index for a given period j is higher than that of a given sequence q), altogether:

$$E[\hat{\delta}_s|\dddot{X}'_{ij}, P_j] = \sum_{i=1}^{I}\sum_{j=2}^{J-1} \lambda_{[\delta_s]ij}(I(j > i)\xi_j))$$
$$= \sum_{j=2}^{J-1}\sum_{i=1}^{j-1} \lambda_{[\delta_s]ij}\xi_j\,.$$

This expression is equivalent for scenarios where multiple $I/Q > 1$ clusters are equally allocated to each sequence q, where we instead have sequence-specific weights $\lambda_{[\delta_s]qj}$ summed over sequences $q \in \{1, ..., j-1\}$ (rather than cluster-specific weights summed over clusters i). Accordingly:

$$E[\hat{\delta}_s|\dddot{X}'_{ij}, P_j] = \sum_{j=2}^{J-1}\sum_{q=1}^{j-1} \lambda_{[\delta_s]qj}\xi_j\,.$$

With all the individual ETI estimators $\hat{\delta}_s$ being shown to be weighted sums of the CTI estimands, we can then demonstrate that the ETATE estimator can also be written as a weighted sum of the CTI estimands:

$$E[\widehat{ETATE}|\dddot{X}'_{ij}, P_j] = \frac{\sum_{s}^{J-1} E[\hat{\delta}_s|\dddot{X}'_{ij}, P_j]}{J-1}$$
$$= \frac{\sum_{s=1}^{J-1}\sum_{j=2}^{J-1}\sum_{q=1}^{j-1}\lambda_{[\delta_s]qj}\xi_j}{J-1}$$
$$= \sum_{j=2}^{J-1} w_3(Q,\gamma,j)\xi_j$$

with estimand weights $w_3(Q,\gamma,j) = \frac{\sum_{s=1}^{J-1}\sum_{q=1}^{j-1}\lambda_{[\delta_s]qj}}{J-1}$, total number of sequences $Q = J-1$, and $\gamma = \frac{\tau_\alpha^2}{\tau_\alpha^2+\tau_\omega^2+\sigma_e^2/K}$ assuming equal cluster-period size K and a nested exchangeable correlation structure.

Altogether, we demonstrate that the ETATE estimator can be written as a weighted average of the CTI estimands (with weights $w_3(Q,\gamma,j)$) in a simple and standard Q sequence and J period SW-CRT design, where there are I/Q clusters in each randomization sequence q. □

C.2 Derivation of the ETATE estimator when the treatment effect varies with calendar time in a 3 sequence, 4 period SW-CRT design

We demonstrate the derivation outlined in Appendix Section C.1 with a $Q = 3$ sequence, $J = 4$ period SW-CRT design. Again, we start the derivation assuming I clusters, with $I/Q = 1$ cluster equally allocated into each sequence q. Accordingly:

$$E[\hat{\delta}_s|\dddot{X}'_{ij}, P_j] = \sum_{j=2}^{3}\sum_{i=1}^{j-1}\lambda_{[\delta_s]ij}\xi_j$$

$$= \lambda_{[\delta_s]12}\xi_2 + \lambda_{[\delta_s]13}\xi_3 + \lambda_{[\delta_s]23}\xi_3$$

Altogether, we can derive and demonstrate that the expected value of the ETATE estimator, given that the true treatment effect structure has calendar time-varying treatment effects is equal to:

$$E[\widehat{ETATE}|\dddot{X}'_{ij}, P_j] = \frac{\sum_{s=1}^{J-1} E[\hat{\delta}_s|\dddot{X}'_{ij}, P_j]}{J-1}$$
$$= \frac{E[\hat{\delta}_1+\hat{\delta}_2+\hat{\delta}_3|\dddot{X}'_{ij}, P_j]}{3}$$
$$= \frac{\sum_{s=1}^{3}(\lambda_{[\delta_s]12})\xi_2 + \sum_{s=1}^{3}(\lambda_{[\delta_s]13}+\lambda_{[\delta_s]23})\xi_3}{3}$$
$$= w_3(Q,\gamma,2)\xi_2 + w_3(Q,\gamma,3)\xi_3$$

where $w_3(Q,\gamma,j)$ are the CTI estimand-specific weights. Analytically solving for these weights $w_3(Q,\gamma,j)$

results in:

$$E[\widehat{ETATE}|\dddot{X}'_{ij}, P_j] = \frac{-9\gamma^2 + 30\gamma + 12}{2(9\gamma^2 + 39\gamma + 13)}\xi_2 + \frac{27\gamma^2 + 48\gamma + 14}{2(9\gamma^2 + 39\gamma + 13)}\xi_3$$

where $\gamma = \frac{\tau_\alpha^2}{\tau_\alpha^2 + \tau_\omega^2 + \sigma_e^2/K}$ (with a nested exchangeable correlation structure and equal cluster-period size K).

D Appendix: Behavior of the CTATE estimator with a true underlying exposure time-varying treatment effect structure

Derivation of the CTATE estimator when the treatment effect varies with exposure time.

The CTI mixed effects model:

$$Y_{ijk} = \dddot{X}'_{ij}\xi + P_j\phi + \alpha_i + \epsilon_{ijk} \, ,$$

can be re-written with matrix notation, using the following design matrix $\dddot{Z} = (\dddot{X}, P)$, and vector of coefficients $\beta_{CTI} = (\xi', \phi')'$:

$$Y = \dddot{Z}\beta_{CTI} + v_{ij} \, .$$

We can specify this with cluster-period means, with Y being the vector of cluster-period mean outcomes $\bar{Y}_{ij}$ and v being the vector of heterogeneity terms $v_{ij} = \alpha_i + \omega_{ij} + \epsilon_{ij}$, where $v \overset{iid}{\sim} N(0, V)$. In such a mixed effects model with a nested exchangeable correlation structure, $V = \mathbb{I}_I \otimes R_i$ is the IJ by IJ block diagonal variance-covariance matrix of Y (where $\mathbb{I}_I$ is an I by I dimension identity matrix) and:

$$R_i = Var(\bar{Y}_{ij})(\mathbb{I}_J(1-\gamma) + \mathbb{J}_j(\gamma))$$

(where $\mathbb{I}_J$ and $\mathbb{J}_J$ are J by J dimension matrices, representing the identity matrix and a matrix of ones, respectively), and where $\otimes$ represents the Kronecker product. With a nested exchangeable correlation structure and fixed cluster-period sizes K, $\gamma = \frac{\tau_\alpha^2}{\tau_\alpha^2+\tau_\omega^2+\sigma_e^2/K}$, and $Var(\bar{Y}_{ij}) = Var(v_{ij}) = \tau_\alpha^2 + \tau_\omega^2 + \sigma_e^2/K$ cancels out of the generalized least squares (GLS) point estimator.

The GLS point estimators for the CTI mixed effects model can accordingly be written as:

$$\widehat{\beta}_{CTI} = (\dddot{Z}'V^{-1}\dddot{Z})^{-1}\dddot{Z}'V^{-1}Y \, .$$

D.1 Derivation of the CTATE estimator when the treatment effect varies with exposure time in a standard Q sequence, J period SW-CRT design

Here, we outline how the CTATE estimator can be written as a weighted average of the ETI estimands in a simple and standard SW-CRT design with I clusters and J periods, where there is $I/Q = 1$ cluster in each randomization sequence $q \in \{1, ..., Q\}$ and $I = J - 1$. This yields an equivalent estimator to scenarios with Q sequences with equal allocation of $I/Q > 1$ clusters randomized into each sequence q.

We must first derive the CTI point estimators $\hat{\xi}_j$ where $j \in \{2, ..., J-1\}$. Notably, $\hat{\xi}_J$ and $\hat{\phi}_J$ from the final period $j = J$ is unidentifiable. Importantly, observations from period J are accordingly excluded from this analysis due to all clusters being exposed to the treatment during this period. To derive these CTI point

estimators, we first derive $(\dddot{Z}'V^{-1}\dddot{Z})$ with block matrices, such that:

$$(\dddot{Z}'V^{-1}\dddot{Z}) = \frac{1}{\sigma^2 + \tau_\alpha^2 + \tau_\omega^2}\begin{pmatrix}\Omega_{11} & \Omega_{12} \\ \Omega_{21} & \Omega_{22}\end{pmatrix}$$

where $\sigma^2 = \frac{\sigma_e^2}{K}$. Here, Ω_{11} is the top-left $(J-2)$ by $(J-2)$ block matrix corresponding with the CTI effects, and Ω_{22} is the bottom-right $(J-1)$ by $(J-1)$ matrix corresponding with the time period effects. Crucially, $(\dddot{Z}'V^{-1}\dddot{Z})$ using information from observed cluster-period cells where $j \neq J$ is a positive-definite matrix; hence we can define:

$$(\dddot{Z}'V^{-1}\dddot{Z})^{-1} = (\sigma^2 + \tau_\alpha^2 + \tau_\omega^2)\begin{pmatrix}\Omega_{11} & \Omega_{12} \\ \Omega_{21} & \Omega_{22}\end{pmatrix}^{-1}$$

The first J-2 rows of $(\dddot{Z}'V^{-1}\dddot{Z})^{-1}$ corresponding to the CTI effects is equal to:

$$(\dddot{Z}'V^{-1}\dddot{Z})^{-1}_{[\xi_2,\ldots,\xi_{J-1}]} = (\sigma^2 + \tau_\alpha^2 + \tau_\omega^2)\begin{pmatrix}(\Omega_{11} - \Omega_{12}\Omega_{22}^{-1}\Omega_{21})^{-1} & -(\Omega_{11} - \Omega_{12}\Omega_{22}^{-1}\Omega_{21})^{-1}\Omega_{12}\Omega_{22}^{-1}\end{pmatrix}$$

Subsequently, $(\dddot{Z}'V^{-1}\dddot{Z})^{-1}_{[\xi_2,\ldots,\xi_{J-1}]}\dddot{Z}'V^{-1}$ is the $(J-2)$ by $(J-1)^2$ matrix:

$$(\dddot{Z}'V^{-1}\dddot{Z})^{-1}_{[\xi_2,\ldots,\xi_{J-1}]}\dddot{Z}'V^{-1} = \begin{pmatrix}\lambda_{[\xi_2]} \\ \vdots \\ \lambda_{[\xi_{J-1}]}\end{pmatrix}$$

where $\lambda_{[\xi_j]}$ is the row vector corresponding to coefficient ξ_j in $(\dddot{Z}'V^{-1}\dddot{Z})^{-1}_{[\xi_2,\ldots,\xi_{J-1}]}\dddot{Z}'V^{-1}$, with $(J-1)^2$ entries $\lambda_{[\xi_j]ij}$ corresponding to cluster-period mean observations from period $j \in \{1, ..., J-1\}$ of cluster $i \in \{1, ..., I\}$ where $I = J - 1$.

Finally, $\hat{\xi}$, the resulting vector of the CTI estimators is:

$$\hat{\xi} = (\dddot{Z}'V^{-1}\dddot{Z})^{-1}_{[\xi_2,\ldots,\xi_{J-1}]}\dddot{Z}'V^{-1}Y\,.$$

Recall that Y is the vector of cluster-period cell means $\bar{Y}_{ij}$. As a result, we can write each $\hat{\xi}_{j=c}$ $\forall c \in \{2, ..., J-1\}$ as:

$$\hat{\xi}_{j=c} = \sum_{i=1}^{I}\sum_{j=1}^{J-1}\lambda_{[\xi_{j=c}]ij}\bar{Y}_{ij}\,.$$

Assuming the underlying marginal model has exposure time-varying treatment effects, such that:

$$E[\bar{Y}_{ij}|\ddot{X}'_{ij}, P_j] = \ddot{X}'_{ij}\delta + P_j\phi$$

is true, the conditional expectation of the CTI estimator $\hat{\xi}_{j=c}$, given covariates $\ddot{X}'_{ij}$ for the true ETI treatment effect structure is then:

$$\begin{aligned}E[\hat{\xi}_{j=c}|\ddot{X}'_{ij}, P_j] &= \sum_{i=1}^{I}\sum_{j=1}^{J-1} \lambda_{[\xi_{j=c}]ij} E[\bar{Y}_{ij}|\ddot{X}'_{ij}, P_j] \\ &= \sum_{i=1}^{I}\sum_{j=1}^{J-1} \lambda_{[\xi_{j=c}]ij} (\ddot{X}'_{ij}\delta + P_j\phi)\,.\end{aligned}$$

In Kenny et al. [3], the following equality was shown to hold:

$$\sum_{i=1}^{I}\sum_{j=1}^{J-1} \lambda_{[\xi_{j=c}]ij} (P_j\phi) = 0$$

$\forall c \in \{2, ..., J-1\}$. Therefore, (1.) we only sum over periods $j \in [2, J-1]$ that have some cluster-period cells receiving the control and others receiving the treatment. Subsequently, we observe that within these periods, only clusters $i \in [1, j-1]$ receive the treatment. Furthermore, (2.) cluster-period cells receiving the treatment during period j of cluster i will receive the exposure-time varying treatment δ_{j-1}. Therefore, we can rewrite $\ddot{X}'_{ij}\delta = I(j > i)\delta_{j-i}$ (with $I(j > i)$ referring to an indicator for whether the index for a given period j is higher than that of a given sequence q). Altogether:

$$\begin{aligned}E[\hat{\xi}_{j=c}|\ddot{X}'_{ij}, P_j] &= \sum_{i=1}^{I}\sum_{j=2}^{J-1} \lambda_{[\xi_{j=c}]ij} (I(j > i)\delta_{j-i}) \\ &= \sum_{j=2}^{J-1}\sum_{i=1}^{j-1} \lambda_{[\xi_{j=c}]ij} \delta_{j-i}\end{aligned}$$

This is equivalent for scenarios where multiple $I/Q > 1$ clusters are equally allocated to each sequence q, where we instead have sequence-specific weights $\lambda_{[\xi_j]qj}$, summed over sequences $q \in \{1, ..., j-1\}$ (rather than cluster-specific weights and summed over clusters i), with observations in sequence q and period j receiving ETI effect δ_{j-q} given that $j > q$. Accordingly:

$$E[\hat{\xi}_{j=c}|\ddot{X}'_{ij}, P_j] = \sum_{j=2}^{J-1}\sum_{q=1}^{j-1} \lambda_{[\xi_{j=c}]qj} \delta_{j-q}$$

With all the individual CTI estimators $\hat{\xi}_j$ being shown to be weighted sums of the ETI estimands, we can then demonstrate that the CTATE estimator can also be written as a weighted sum of the ETI estimands:

$$\begin{aligned}E[\widehat{CTATE}|\ddot{X}'_{ij}, P_j] &= \frac{\sum_{c=2}^{J-1} E[\hat{\xi}_{j=c}|\ddot{X}'_{ij}, P_j]}{J-2} \\ &= \frac{\sum_{c=2}^{J-1}\sum_{j=2}^{J-1}\sum_{q=1}^{j-1} \lambda_{[\xi_{j=c}]qj} \delta_{j-q}}{J-2}\,.\end{aligned}$$

Writing the expression in terms of exposure time s, we have:

$$E[\widehat{CTATE}|\ddot{X}'_{ij}, P_j] = \frac{\sum_{s=1}^{J-2}(\sum_{c=2}^{J-1}\sum_{j=2}^{J-1}\sum_{q=1}^{J-1} I(j-q=s)\lambda_{[\xi_{j=c}]qj})\delta_s}{J-2}$$

$$= \sum_{s=1}^{J-2} w_4(Q,\gamma,s)\delta_s$$

with estimand weights $w_4(Q,\gamma,s) = \frac{\sum_{c=2}^{J-1}\sum_{j=2}^{J-1}\sum_{q=1}^{J-1} I(j-q=s)\lambda_{[\xi_{j=c}]qj}}{J-2}$ (with $I(j-q=s)$ referring to an indicator for indices where the difference between the given period j and sequence q is equal to the given exposure time s, $j-q=s$), total number of sequences $Q = J-1$, and $\gamma = \frac{\tau_\alpha^2}{\tau_\alpha^2+\tau_\omega^2+\sigma_e^2/K}$ (assuming equal cluster-period size K and a nested exchangeable correlation structure).

Altogether, we demonstrate that the CTATE estimator can be written as a weighted average of the ETI estimands (with weights $w_4(Q,\gamma,s)$) in a simple and standard Q sequence and J period SW-CRT design, where there are I/Q clusters in each randomization sequence q. □

D.2 Derivation of the CTATE estimator when the treatment effect varies with exposure time in a 3 sequence, 4 period SW-CRT design

We demonstrate the derivation outlined in Appendix Section D.1 with a $Q = 3$ sequence, $J = 4$ period SW-CRT design. Again, we start the derivation assuming I clusters with $I/Q = 1$ cluster equally allocated into each sequence q. Accordingly:

$$E[\hat{\xi}_{j=c}|\ddot{X}'_{ij}, P_j] = \sum_{j=2}^{3}\sum_{i=1}^{j-1} \lambda_{[\xi_{j=c}]ij}\delta_{j-i}$$

$$= \lambda_{[\xi_{j=c}]12}\delta_1 + \lambda_{[\xi_{j=c}]13}\delta_2 + \lambda_{[\xi_{j=c}]23}\delta_1$$

Altogether, we can derive and demonstrate that the expected value of the CTATE estimator, given that the true treatment effect structure has exposure time-varying treatment effects is equal to:

$$E[\widehat{CTATE}|\ddot{X}'_{ij}, P_j] = \frac{\sum_{c=2}^{J-1} E[\hat{\xi}_{j=c}|\ddot{X}'_{ij}, P_j]}{J-2}$$

$$= \frac{E[\hat{\xi}_2 + \hat{\xi}_3|\ddot{X}'_{ij}, P_j]}{2}$$

$$= \frac{\sum_{j=2}^{3}(\lambda_{[\xi_{j=c}]12} + \lambda_{[\xi_{j=c}]23})\delta_1 + \sum_{j=2}^{3}(\lambda_{[\xi_{j=c}]13})\delta_2}{2}$$

$$= w_4(Q,\gamma,1)\delta_1 + w_4(Q,\gamma,2)\delta_2$$

where $w_4(Q,\gamma,s)$ are the ETI estimand-specific weights corresponding to exposure time s. Analytically

solving for these weights $w_4(Q, \gamma, s)$ results in:

$$E[\widehat{CTATE}|\ddot{X}'_{ij}, P_j] = \frac{9\gamma^2 + 15\gamma + 6}{2(3\gamma^2 + 8\gamma + 4)}\delta_1 + \frac{-3\gamma^2 + \gamma + 2}{2(3\gamma^2 + 8\gamma + 4)}\delta_2$$

where $\gamma = \frac{\tau_\alpha^2}{\tau_\alpha^2 + \tau_\omega^2 + \sigma_e^2/K}$ (with a nested exchangeable correlation structure and equal cluster-period size K).

E Appendix: Complete scaled weight graphs of misspecified estimators

Scaled weights of misspecified estimators are graphed across numbers of periods and values of γ.

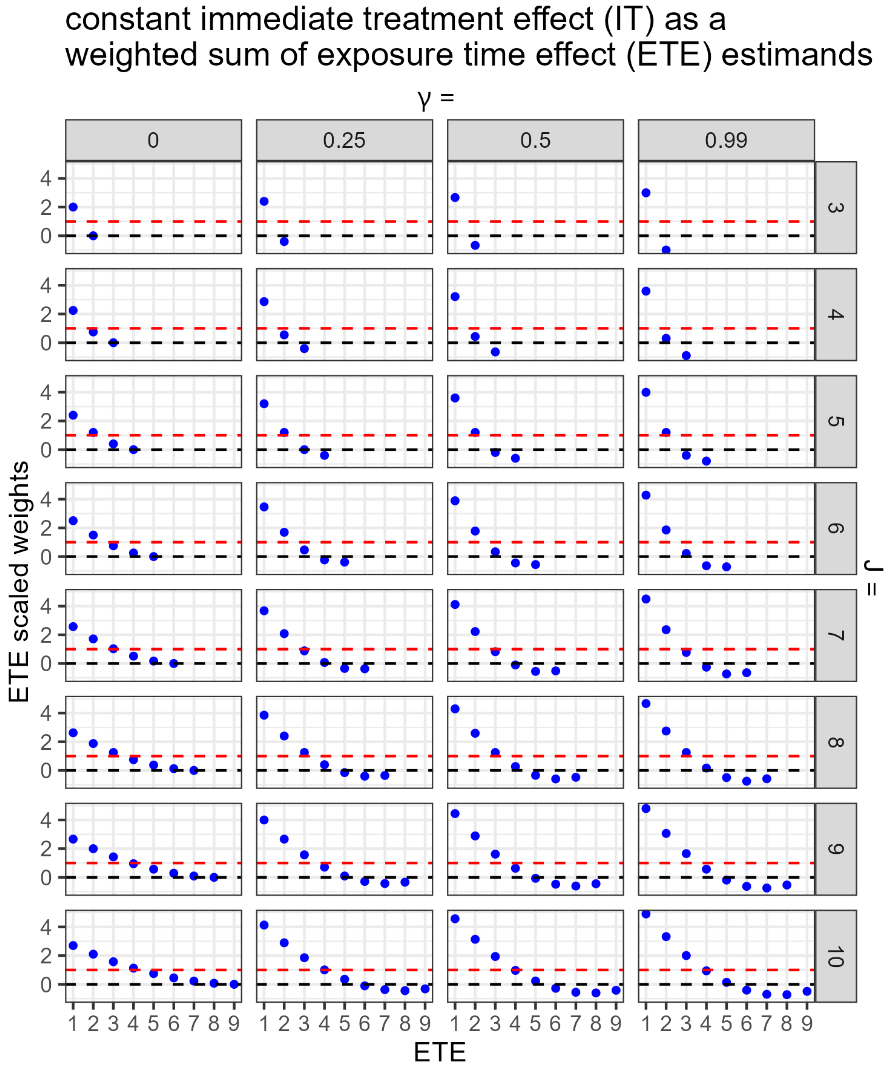

Exposure time-varying treatment effects and their corresponding scaled weights $(J-1)[w_1(Q,\gamma,s)]$ in the IT estimator, graphed on the x-axis and y-axis, respectively. Results are presented across SW-CRTs with different total numbers of periods J and varying γ ($\gamma = \frac{\tau_\alpha^2}{\tau_\alpha^2+\tau_\omega^2+\sigma_e^2/K}$ with a nested exchangeable correlation structure). Dotted red line marks a nominal weight of 1. Dotted black line marks a weight of 0.

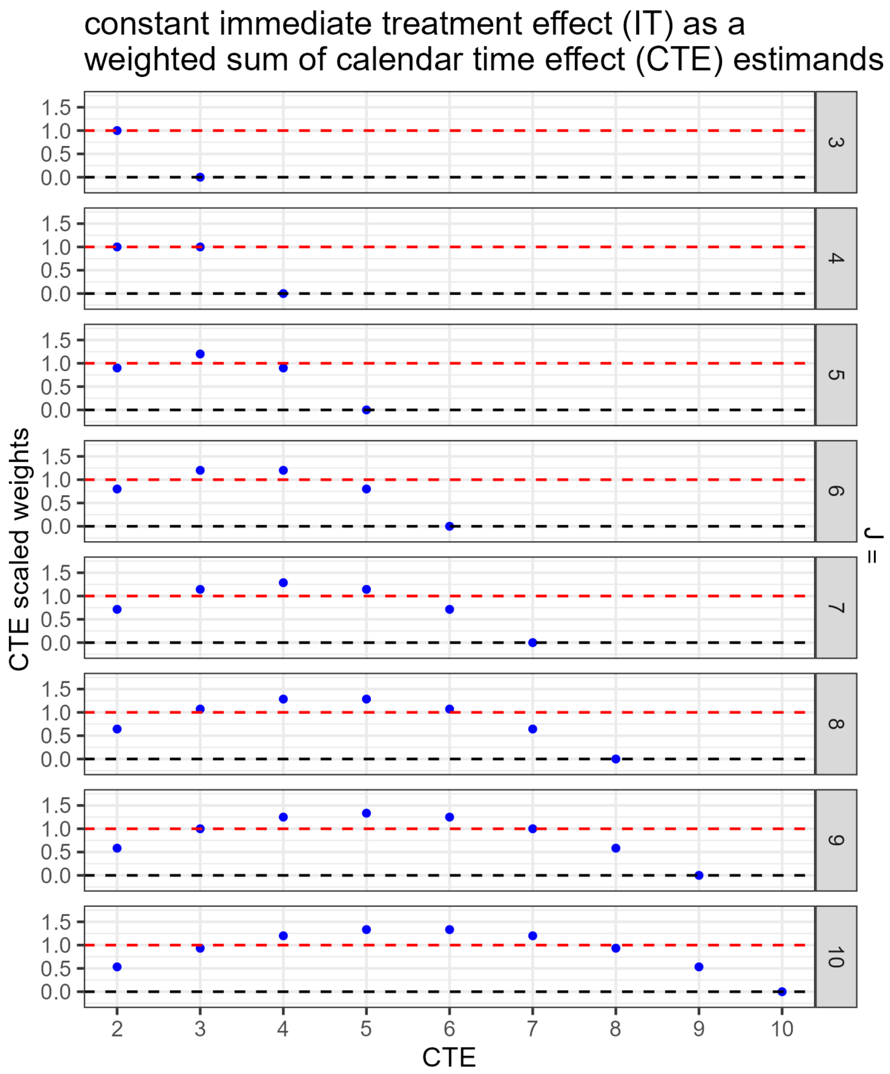

Calendar time-varying treatment effects and their corresponding scaled weights $(J-2)[w_2(Q,j)]$ in the IT estimator, graphed on the x-axis and y-axis, respectively. Results are presented across SW-CRTs with different total numbers of periods J. Dotted red line marks a nominal weight of 1. Dotted black line marks a weight of 0.

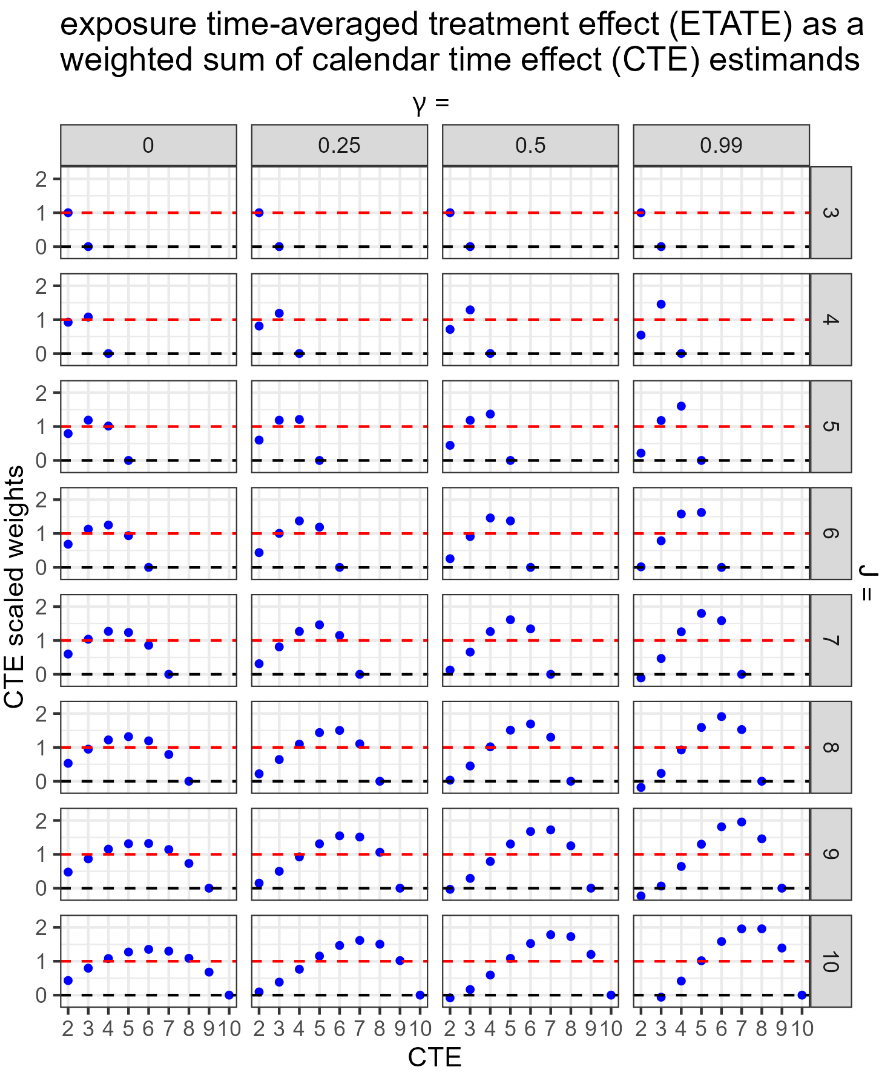

Calendar time-varying treatment effects and their corresponding scaled weights $(J-2)[w_3(Q,\gamma,j)]$ in the ETATE estimator, graphed on the x-axis and y-axis, respectively. Results are presented across SW-CRTs with different total numbers of periods J and varying γ ($\gamma = \frac{\tau_\alpha^2}{\tau_\alpha^2+\tau_\omega^2+\sigma_e^2/K}$ with a nested exchangeable correlation structure). Dotted red line marks a nominal weight of 1. Dotted black line marks a weight of 0.

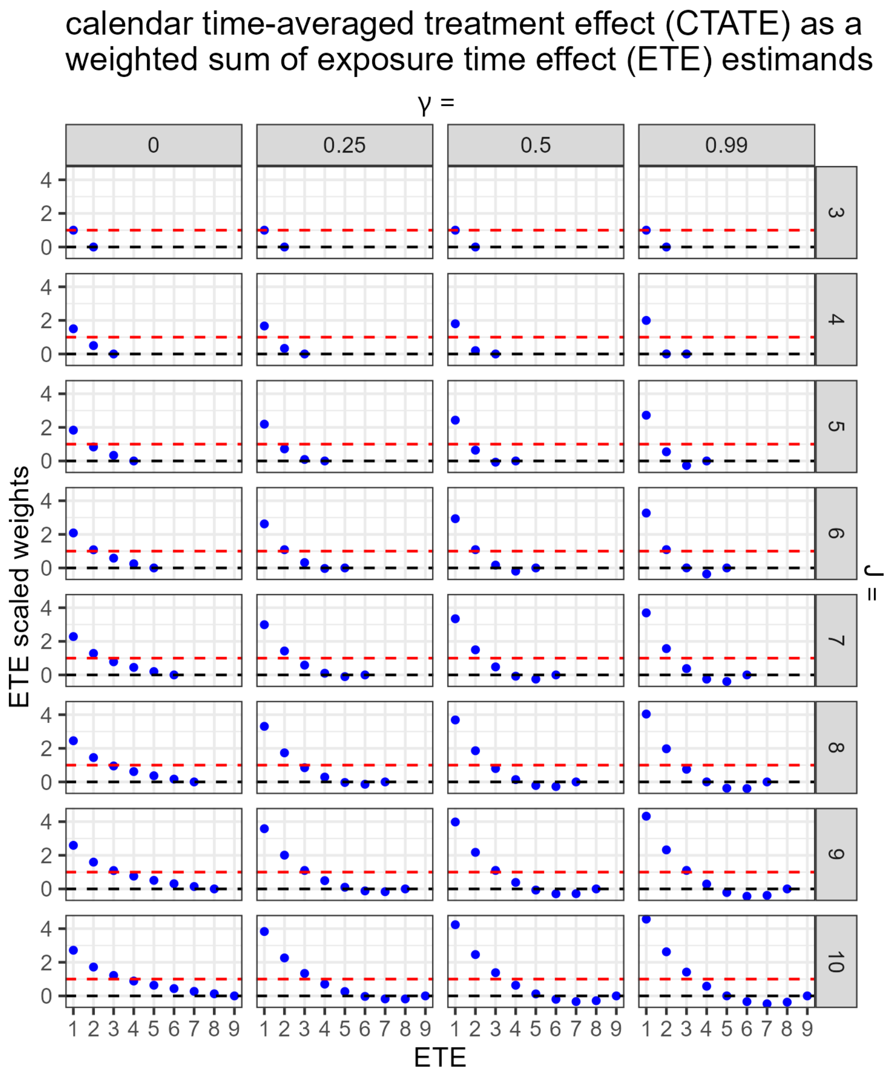

Exposure time-varying treatment effects and their corresponding scaled weights $(J-2)w_4(Q,\gamma,s)$ in the CTATE estimator, graphed on the x-axis and y-axis, respectively. Results are presented across SW-CRTs with different total numbers of periods J and varying γ ($\gamma = \frac{\tau_\alpha^2}{\tau_\alpha^2+\tau_\omega^2+\sigma_e^2/K}$ with a nested exchangeable correlation structure). Dotted red line marks a nominal weight of 1. Dotted black line marks a weight of 0.

F Appendix: Additional Scenarios with CTE as analyzed by an IT estimator

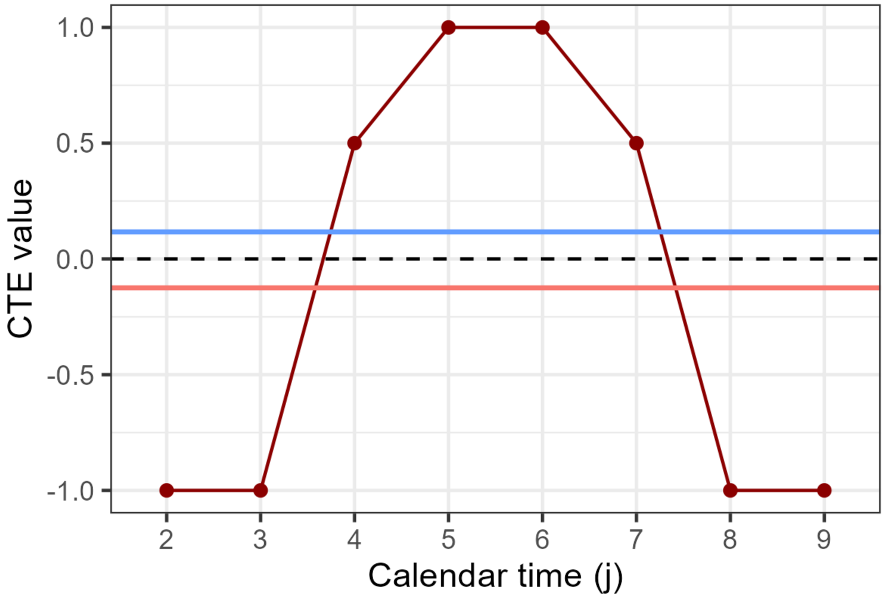

An additional scenarios with different calendar time-varying treatment effects (CTE) as analyzed by an immediate treatment effect (IT) estimator in a 9 cluster, 10 period SW-CRT. CTE are only identifiable up to time period 9 with the inclusion of period fixed effects. The corresponding IT and true CTATE effect estimates are shown in the blue and red lines, respectively. Dotted black line marks an effect of 0.

G Appendix: Additional Simulation results

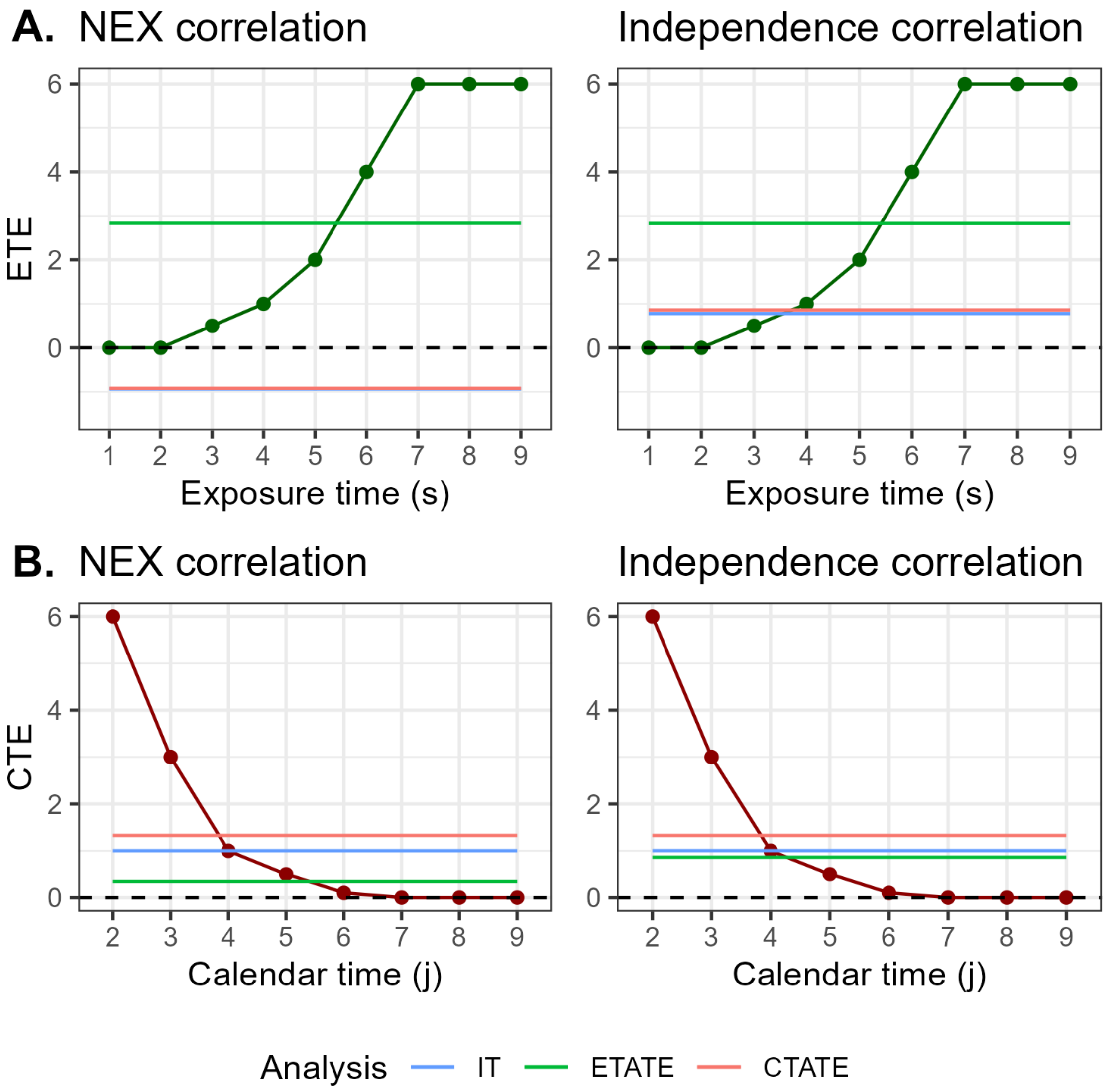

Simulated (A.) exposure time-varying treatment effect (ETE) curve and (B.) calendar time-varying treatment effect (CTE) in a 18 cluster, 10 period SW-CRT with an average cluster-period cell size of 30 individuals. Results are derived with either a nested exchangeable (NEX) or independence correlation structure. Corresponding immediate treatment effect (IT), exposure time-averaged treatment effect (ETATE), calendar time-averaged treatment effect (CTATE) estimates are plotted as horizontal lines. Analysis results are summarized over 1000 simulated replicates.

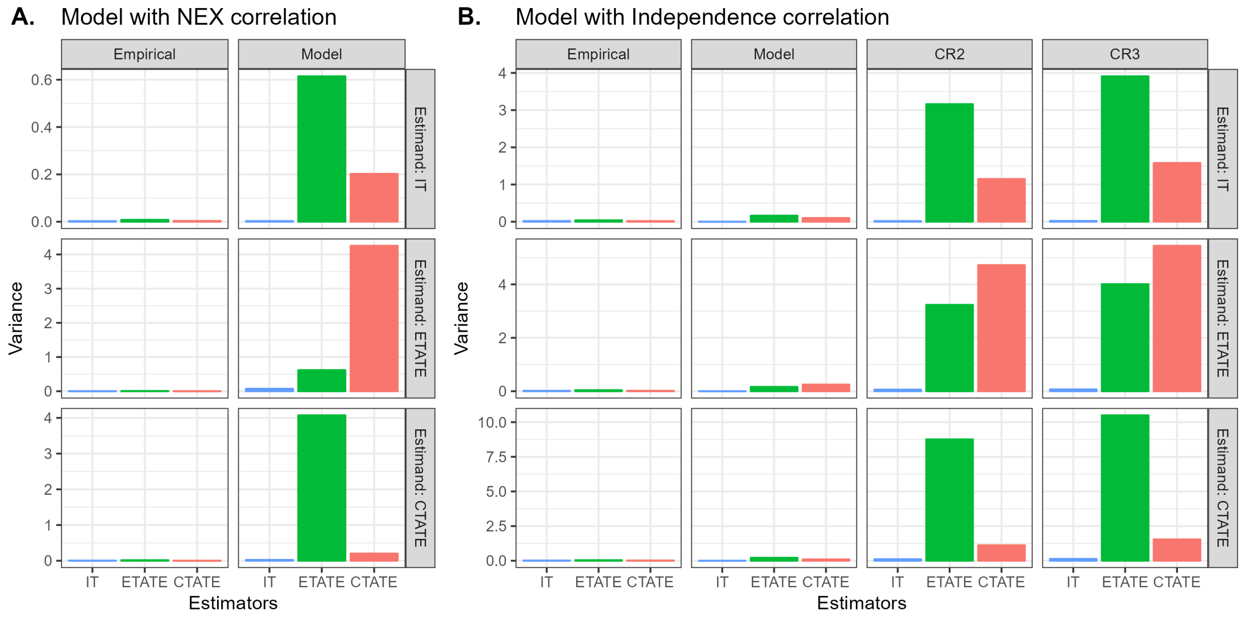

Simulation efficiency results, as shown with empirical variances (Empirical) being the variance of the point estimator over 1000 simulation replicates, are plotted alongside the average of model-based variance estimates (Model) for calendar time-averaged treatment effect (CTATE), exposure time-averaged treatment effect (ETATE), and immediate treatment effect (IT) estimands. Corresponding results from the $\widehat{CTATE}$, $\widehat{ETATE}$, and $\widehat{IT}$ estimators are plotted on the x-axis. Results are plotted for analytic models with either a (A.) nested exchangeable correlation (NEX), or (B.) independence correlation. Since the underlying data-generating process had a nested exchangeable correlation, bias-reduced linearization (CR2) and "approximate jackknife" (CR3) cluster robust variance estimators are also included for analyses with an independence correlation structure.

H Appendix: Additional Case Study Re-Analysis results

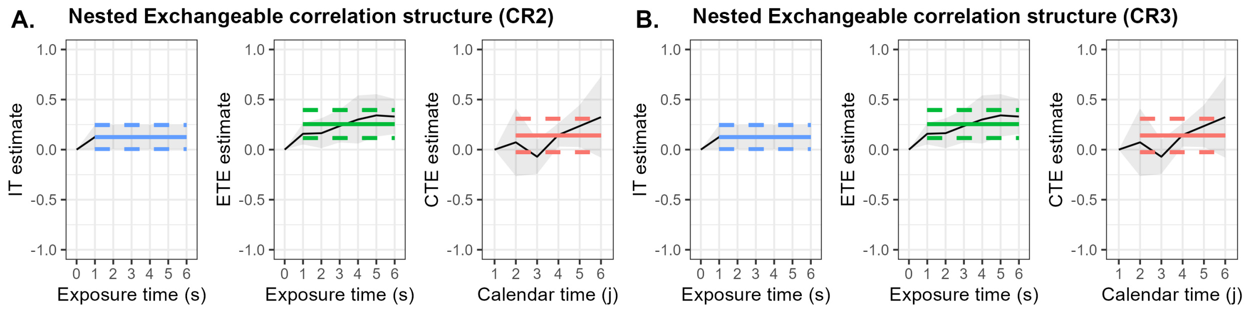

Effect curves over calendar time and exposure time from an Australia disinvestment trial as analyzed with an immediate treatment, exposure time indicators, or calendar time indicators with nested exchangeable correlation structure. 95% confidence intervals of each point on the effect curve are shown in gray using (A.) bias-reduced linearization (CR2) and (B.) "approximate jackknife" (CR3) cluster robust variance estimators. The blue, green, and red solid lines denote the corresponding IT, ETATE, and CTATE estimates, accordingly, with dotted lines denoting the 95% confidence intervals of the IT, ETATE, and CTATE estimates.
